# Influence of attributional style, anger, and self-control on college students’ impulsive consumption

**DOI:** 10.3389/fpsyg.2025.1641481

**Published:** 2025-09-26

**Authors:** Guohua Zhou, Wentao Sun, Weiguo Qu

**Affiliations:** ^1^School of Education Science, Hunan Normal University, Changsha, China; ^2^Cognition and Human Behavior Key Laboratory of Hunan Province, Changsha, China

**Keywords:** college students impulsive consumption, attributional style, anger, self-control, educational interventions

## Abstract

**Introduction:**

With the rapid development of the digital economy and the prevalence of consumerism in recent years, impulsive consumption among college students has become increasingly prominent, a phenomenon closely associated with their psychological traits and emotional states. Even though research has examined self-control and consumption, few studies have integrated attributional style. Therefore, this study systematically examines the effects of attributional style, anger, and self-control on impulsive consumption behaviors among college students through three experiments.

**Methods:**

Undergraduate participants were recruited from a university in Changsha, China. Experiment 1 investigated the impact of attributional style on impulsive consumption. Experiment 2 explored the interactive effects of anger and attributional style on impulsive consumption. Finally, Experiment 3 further examined the interaction between self-control and attributional style.

**Results:**

Experiment 1 found that participants with optimistic attributional styles exhibited significantly lower levels of impulsive consumption than those with pessimistic attributional styles. Experiment 2 revealed that anger significantly suppressed impulsive consumption behaviors and interacted with attributional style. Experiment 3 demonstrated that higher levels of self-control effectively reduced impulsive consumption and produced significant interactive effects with attributional style.

**Discussion:**

These findings indicate that optimistic attributional styles, anger induction, and high self-control significantly inhibit impulsive consumption among college students. These results provide novel theoretical insights into the psychological mechanisms underlying impulsive consumption in this population and offer empirical support for targeted psychological interventions in consumer behavior.

## 1 Introduction

Impulsive consumption resembles a sudden gust of wind—overpowering in the moment, but is often followed by regret. It refers to irrational purchasing decisions made under weakened self-control, owing to strong emotional responses triggered by external or internal stimuli. Among college students, impulsive consumption manifests distinctively through the excessive pursuit of branded products and heightened sensitivity to promotional campaigns (Xie et al., 2023). During purchase decisions, students frequently prioritize rapid emotional reactions over rational deliberation, reflecting both their desire for immediate gratification and their vulnerability to external temptations. Frequent impulsive consumption exacerbates financial strain and adversely affects academic performance and quality of life (Guo et al., 2024). Studies indicate a strong correlation between excessive spending and compromised mental health in this demographic, often leading to anxiety and self-reproach (Rook and Fisher, 1995; Yi and Baumgartner, 2011). Moreover, impulsive consumption perpetuates a vicious cycle by undermining financial management capabilities as individuals neglect self-regulatory strategies (Guo et al., 2024). These findings underscore the need to investigate impulsive consumption among college students.

Attributional style plays a pivotal role in emotional regulation and decision-making (Chen et al., 2025). Defined as an individual’s habitual explanation for events, attributional style is broadly categorized into optimistic and pessimistic types (Weiner, 1985; Sanders, 2024). Optimistic individuals attribute success to internal efforts and failures to external factors, fostering resilience and emotional stability that mitigate impulsive tendencies (Wang et al., 2020). Conversely, those with pessimistic styles tend to internalize failures as personal inadequacies, heightening emotional distress and impulsive behaviors (Antosz-Rekucka and Prochwicz, 2024). College students, often navigating academic pressures and financial constraints, exhibit divergent consumption patterns influenced by attributional styles. Research suggests that optimistic attributional style correlates with stronger self-control, thus enabling rational resistance to consumption temptations (Hofmann et al., 2012a). Understanding these dynamics offers critical insights into the psychological mechanisms of impulsive consumption and can inform targeted interventions. Thus, we hypothesized the following:

*Hypothesis 1:* As attributional style influences impulsive consumption, college students with optimistic attributional style exhibit lower levels of impulsive consumption than those with pessimistic style.

Anger, a fundamental negative emotion, is defined as the intense discomfort arising from conflicts in personal values or perceived rights (Lazarus, 1991; Renström et al., 2023). Characterized by low valence and high arousal (Scherer, 2005), anger is a discrete emotion with distinct cognitive and behavioral signatures. Unlike other negative emotions, anger is transient yet highly activating. Its impact on consumption decisions is complex. While positive emotions generally enhance purchasing behaviors (Herabadi et al., 2009), findings on negative emotions remain inconsistent. Some studies suggest that anger amplifies impulsive consumption by driving individuals toward immediate gratification (Rook, 1987; Verplanken and Herabadi, 2001; Redine et al., 2023), whereas others propose that anger has inhibitory effects (Herabadi et al., 2009). Notably, anger may uniquely suppress impulsive consumption compared with other negative emotions (Lerner and Keltner, 2000). College students with underdeveloped emotional regulation skills (Gross, 2015) and frequent exposure to anger-inducing stressors (e.g., academic competition and financial strain) are particularly vulnerable. Optimistic individuals may externalize anger (e.g., attributing frustration to unethical marketing) and employ rational coping strategies (e.g., resisting purchases), whereas pessimistic individuals exhibit heightened dorsolateral prefrontal cortex activation during anger, reflecting a greater cognitive effort to regulate emotions and suppress impulses (Disner et al., 2011). This interplay between attributional style and anger highlights the need to explore their combined effects on impulsive consumption. Thus, we hypothesized the following:

*Hypothesis 2:* Anger influences impulsive consumption; thus, following anger induction, college students exhibit lower impulsive consumption than those under neutral conditions.

*Hypothesis 3:* Attributional style and anger interact to influence impulsive consumption. Thus, following anger induction, optimistic individuals show the lowest impulsive consumption, whereas pessimistic individuals under neutral conditions exhibit the highest impulsive consumption.

Self-control—a core metacognitive ability—serves as a critical “braking system” in impulsive decision-making (Baumeister et al., 2007). In college students, who face relentless consumption stimuli (e.g., social media marketing and time-limited discounts) but lack financial literacy, self-control directly determines resistance to immediate temptations. It also interacts with emotional regulation; optimistic individuals consider temptations as external and transient (e.g., “marketing traps”), conserving cognitive resources for self-control, whereas pessimistic individuals internalize failures (e.g., “I lack willpower”), depleting self-regulatory capacity (Hofmann et al., 2012b; Wang et al., 2020). Even though research has examined self-control and consumption, few studies have integrated attributional style, a gap that this study addresses to comprehensively unravel the psychological mechanisms of impulsive behavior. Thus, we hypothesized the following:

*Hypothesis 4:* Self-control influences impulsive consumption; thus, students with high self-control exhibit lower impulsive consumption than those with low self-control.

*Hypothesis 5:* Attributional style and self-control interact to influence impulsive consumption; thus, optimistic individuals with high self-control show the lowest impulsive consumption, whereas pessimistic individuals with low self-control exhibit the highest impulsive consumption.

In summary, attributional style, anger, and self-control collectively shape impulsive consumption among college students. In today’s consumerist environment, where marketing tactics relentlessly target this demographic, understanding these interactions is critical for fostering rational consumption habits and enhancing self-regulatory capacities (Xie et al., 2023). This study employs simulated scenarios to systematically investigate these dynamics, offering theoretical and practical implications for mitigating impulsive consumption.

## 2 Experiment 1: Impact of attributional style on college students’ impulsive consumption

### 2.1 Research purpose and hypotheses

This experiment examines the influence of attributional style (optimistic vs. pessimistic) on impulsive consumption among college students by simulating impulsive purchase scenarios. The hypothesis tested by Experiment 1 was as follows:

*Hypothesis 1:* Attributional style significantly affects impulsive consumption; thus, students with pessimistic attributional styles exhibit higher levels of impulsive consumption than those with optimistic styles.

### 2.2 Methodology

#### 2.2.1 Participants

A power analysis conducted via G*Power 3.1 (Faul et al., 2007) indicated that a sample size of 128 participants was required to achieve 80% power (1–β) with a medium effect size (*f* = 0.25) at α = 0.05. Using convenience sampling, we recruited 138 undergraduate students from a university in Changsha, China. After excluding 8 invalid responses (incomplete, duplicate, or inconsistent responses), 130 valid datasets were retained. Participants were classified into optimistic (n = 36) and pessimistic (n = 36) attributional style groups based on the top and bottom 27% scores from the Attributional Style Questionnaire (ASQ). The final sample comprised 72 participants (51 female; mean age: *M*_age_ = 20.11 years, standard deviation: *SD* = 1.181). All participants were physically and mentally healthy with normal or corrected vision, had native Chinese proficiency, were right-handed, had basic computer skills, and had no prior exposure to similar experiments. All signed informed consent forms.

#### 2.2.2 Experimental design

A single-factor, between-subjects design was employed with attributional style (optimistic vs. pessimistic) as the independent variable (categorized via ASQ total scores) and impulsive consumption levels as the dependent variable.

#### 2.2.3 Experimental materials

##### 2.2.3.1 ASQ

This study employed the ASQ developed by Peterson and Seligman (1984) and revised by Wang and Zhang (2006). The revised version was adapted to better align with the Chinese cultural context and national conditions, thereby ensuring its applicability to the Chinese student population. Based on the analytical results of this study, the questionnaire demonstrated good internal consistency with a Cronbach’s α coefficient of 0.85. The ASQ comprises 12 hypothetical situations: 6 positive and 6 negative, with 3 questions per situation covering diverse aspects of daily life and work. Responses are recorded on a 7-point Likert scale. For positive situations, 1 represents the least optimistic attribution and 7 the most optimistic, whereas the scoring is reversed for negative situations; scores for positive attribution style and negative attribution style are calculated by dividing the total scores of positive and negative situations by 6, respectively. Higher scores indicate stronger attributional tendencies in the corresponding domains. The overall attribution style is derived by subtracting the negative attribution style score from the positive one. Specifically, an overall attribution style score above 0 signifies an optimistic attribution style, below 0 signifies a pessimistic style, and 0 indicates a neutral attribution style (Yang et al., 2021). In the subsequent experiments, participants from the top and bottom 27% of the ASQ score distribution were selected to form distinct optimistic and pessimistic groups. This extreme-groups approach is a well-established methodological practice in individual differences research (Preacher et al., 2005) that is employed to enhance statistical power for detecting hypothesized effects between theoretically distinct groups by maximizing between-group variance. Notably, this approach excludes mid-scoring participants and may consequently inflate effect size estimates compared to a full-spectrum analysis; therefore, the findings should be interpreted as reflecting differences between relatively pure, extreme groups.

##### 2.2.3.2 Impulsive consumption scenario simulation

This experiment adopted the “simulated scenario” method (Rook and Fisher, 1995) to investigate impulsive consumption (for similar approaches, see Zhou et al., 2017; Song et al., 2021; Xue et al., 2023). Participants were instructed to imagine their shopping decisions and consumption behaviors in hypothetical third-person virtual scenarios. This method was selected not only for its strong experimental control, but also for its ability to standardize stimuli across participants and prevent ethical and practical concerns (e.g., real financial incentives or deception). While acknowledging that hypothetical scenarios may not fully capture the emotional arousal associated with real-world spending, this approach has been widely validated in impulse buying literature and has demonstrated good predictive validity for assessing behavioral tendencies. Specifically, the experimental design included three distinct shopping scenarios, each offering four response options, to measure participants’ impulsive consumption levels. In each scenario, options were structured to reflect varying degrees of impulsive consumption characteristics, with higher scores indicating stronger impulsive tendencies. This method is widely utilized in studies of individual impulsive consumption behavior owing to its efficacy in capturing decision-making processes within controlled contexts. The simulation of impulsive consumption scenarios was conducted online using E-Prime 2.0 software to ensure procedural standardization and accurate data collection.

#### 2.2.4 Procedure

Prior to the formal experiment, participants were required to complete the ASQ. Based on their total ASQ scores, they were divided into an optimistic attribution style group (top 27%, n = 36) and a pessimistic attribution style group (bottom 27%, n = 36). Participants in both groups were then assigned to separate computer laboratories to ensure environmental independence and procedural control.

**Figure 1:**
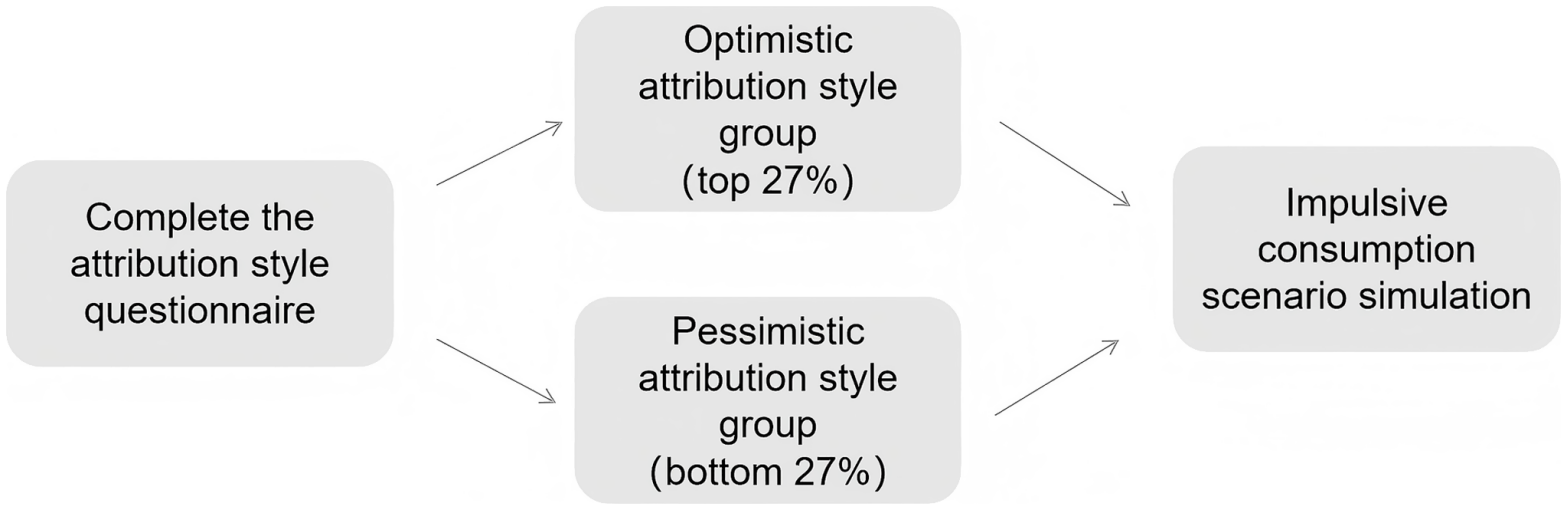
Experimental procedure of Experiment 1.

During the formal experiment, participants in the optimistic and pessimistic attribution style groups were individually guided to complete the impulsive consumption scenario simulation on computers. Each participant responded to three distinct types of shopping decision-making tasks, with their scores directly reflecting their level of impulsive consumption. This was approved by the ethics committee (Figure 1).

#### 2.2.5 Data analysis

All the statistical analyses were performed using SPSS 27.0 (IBM Corp., Armonk, NY, USA). Independent samples t-tests were used to compare the levels of impulsive consumption between participants with different attributional styles (optimistic vs. pessimistic).

### 2.3 Results

**Figure 2:**
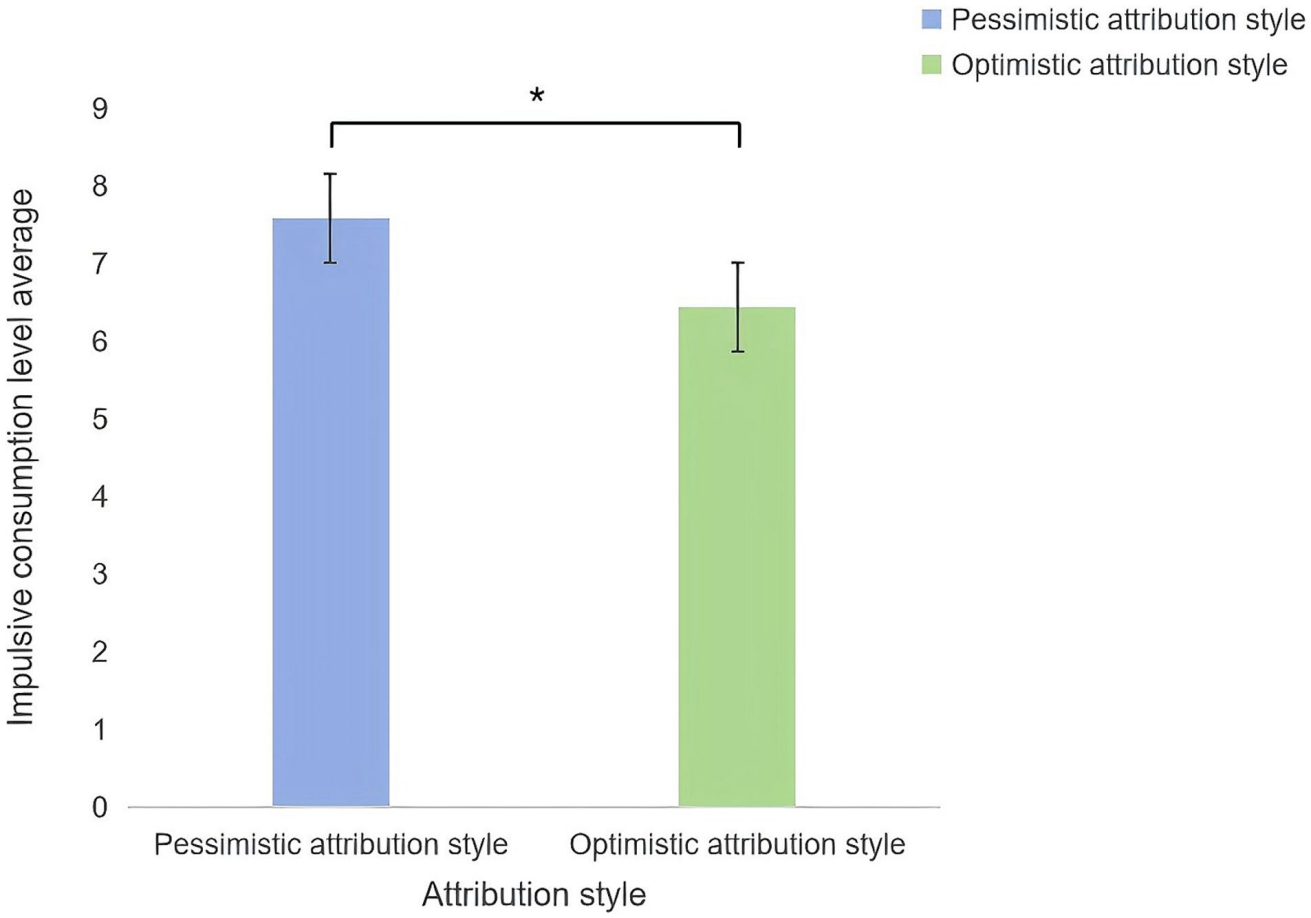
Differences in impulse consumption levels among different attribution styles.

The independent-samples t-test revealed significant differences in impulsive consumption between the attributional style groups (t(1,70) = 2.25, *p* = 0.027, 95% [CI] (0.13, 2.15)). Participants with pessimistic styles (*M* = 7.58, *SD* = 2.34) exhibited higher impulsive consumption than those with optimistic styles (*M* = 6.44, *SD* = 1.93; Figure 2).

### 2.4 Discussion

The results of Experiment 1 demonstrated a significant influence of attributional style on impulsive consumption levels among college students. Analysis indicated marked differences in impulsive consumption, with the optimistic group exhibiting significantly lower levels than the pessimistic group, consistent with Hypothesis 1. This disparity may stem from the optimistic group’s broader information-gathering scope and higher-quality decision-making processes, which likely predisposed them to more rational consumption behaviors (Wang and Liu, 2008).

Research has identified emotional factors alongside attributional style as critical determinants of impulsive consumption (Loewenstein, 2000; Verhagen and van Dolen, 2011). However, the role of anger, a salient emotional factor that shapes impulsive consumption among university students, remains underexplored. To address this gap, Experiment 2 introduced anger induction to investigate the interplay between attributional style, anger, and impulsive consumption. Participants with differing attributional styles engaged in simulated impulsive consumption scenarios under controlled anger conditions, enabling an examination of how these variables jointly influence decision-making.

## 3 Experiment 2: Effects of attributional style and anger on college students’ impulsive consumption

### 3.1 Research purpose and hypotheses

This experiment explored the influence of attribution style and anger on college students’ impulsive consumption by inducing anger and simulating impulsive consumption scenarios in participants with different attribution styles. Accordingly, the following hypotheses were proposed:

*Hypothesis 2:* Anger significantly influences impulsive consumption; thus, students under neutral conditions exhibit higher impulsive consumption than those following anger induction.

*Hypothesis 3:* Attributional style and anger interact to influence impulsive consumption. Thus, under neutral conditions, pessimistic individuals show the highest impulsive consumption, whereas following anger induction, optimistic individuals exhibit the lowest impulsive consumption.

### 3.2 Methodology

#### 3.2.1 Participants

A power analysis (G*Power 3.1; Faul et al., 2007) determined a required sample size of 201 participants for 80% power (1–β) with α = 0.05 and a medium effect size (*f* = 0.25). Using convenience sampling, 234 undergraduate students were recruited from a university in Changsha, China. After excluding 11 invalid responses (incomplete or inconsistent answers), 223 valid datasets were retained. Participants who scored in the top and bottom 27% of the ASQ score were classified as optimistic (n = 60) or pessimistic (n = 60), respectively. Each group was further divided into anger induction (n = 30) and neutral (n = 30) conditions, yielding 120 participants (59 female; *M*_age_ = 17.9 years, *SD* = 0.53). All participants met the same health, linguistic, and procedural criteria as in Experiment 1. All signed informed consent forms.

#### 3.2.2 Experimental design

A 2 (attributional style: optimistic versus pessimistic) × 2 (anger: induced versus neutral) between-subjects design was employed. The independent variables included attributional style (categorized by ASQ scores) and anger (induced by validated video stimuli). The dependent variable was impulsive consumption level.

#### 3.2.3 Experimental materials

##### 3.2.3.1 ASQ

These were the same as those employed in Experiment 1.

##### 3.2.3.2 Impulsive consumption scenario simulation

These were the same as those employed in Experiment 1.

##### 3.2.3.3 Emotional induction materials

The anger-inducing video clip was obtained from *Nanjing! Nanjing!,* part of the *Chinese Emotional Video System* (Xu et al., 2010). The 4-min-and-49-s segment depicts the atrocities committed by Japanese soldiers against Chinese civilians. Following prior methodologies (Li et al., 2025), anger was targeted as the focal emotion in subsequent analyses. The control group viewed a neutral 5-min segment from *Weather Forecast* that aired on September 1, 2023.

##### 3.2.3.4 Positive and negative affect schedule

The participants’ emotional valence was assessed using the Positive and Negative Affect Schedule (Watson et al., 1988; see Appendix 3). This 5-point Likert scale ranges from 1 (*not at all*) to 5 (*extremely intense*), measuring 7 basic emotional dimensions: pleasure, surprise, fear, disgust, sadness, anger, and calmness. Similar protocols were adopted by Liang et al. (2017) and Wong et al. (2021). Baseline emotional states were recorded before video exposure, followed by post-induction measurements. The questionnaire demonstrated high internal consistency in this study (Cronbach’s α = 0.87).

#### 3.2.4 Procedure

Prior to the formal experiment, all participants completed the ASQ. Based on their total ASQ scores, participants were stratified into two groups: those with optimistic attributional styles (top 27%, n = 60) and those with pessimistic attributional styles (bottom 27%, n = 60). Each group was then randomly subdivided into 2 subgroups of 30 participants each: 1 subgroup received anger induction and the other served as a control. Four experimental conditions were assigned to separate computer laboratories to prevent cross-conditioning interference. The experimenter thoroughly explained the experimental protocol and operational guidelines, emphasizing strict compliance with instructional prompts throughout the procedure.

At the start of the experiment, participants completed the Emotional Self-Assessment Scale as a pretest measure of baseline affective states. For the emotion induction phase, 30 optimistic and 30 pessimistic participants viewed a neutral documentary (“*Weather Forecast*”), whereas 30 optimistic and 30 pessimistic participants viewed an anger-inducing film clip (“*Nanjing! Nanjing!*”).

**Figure 3:**
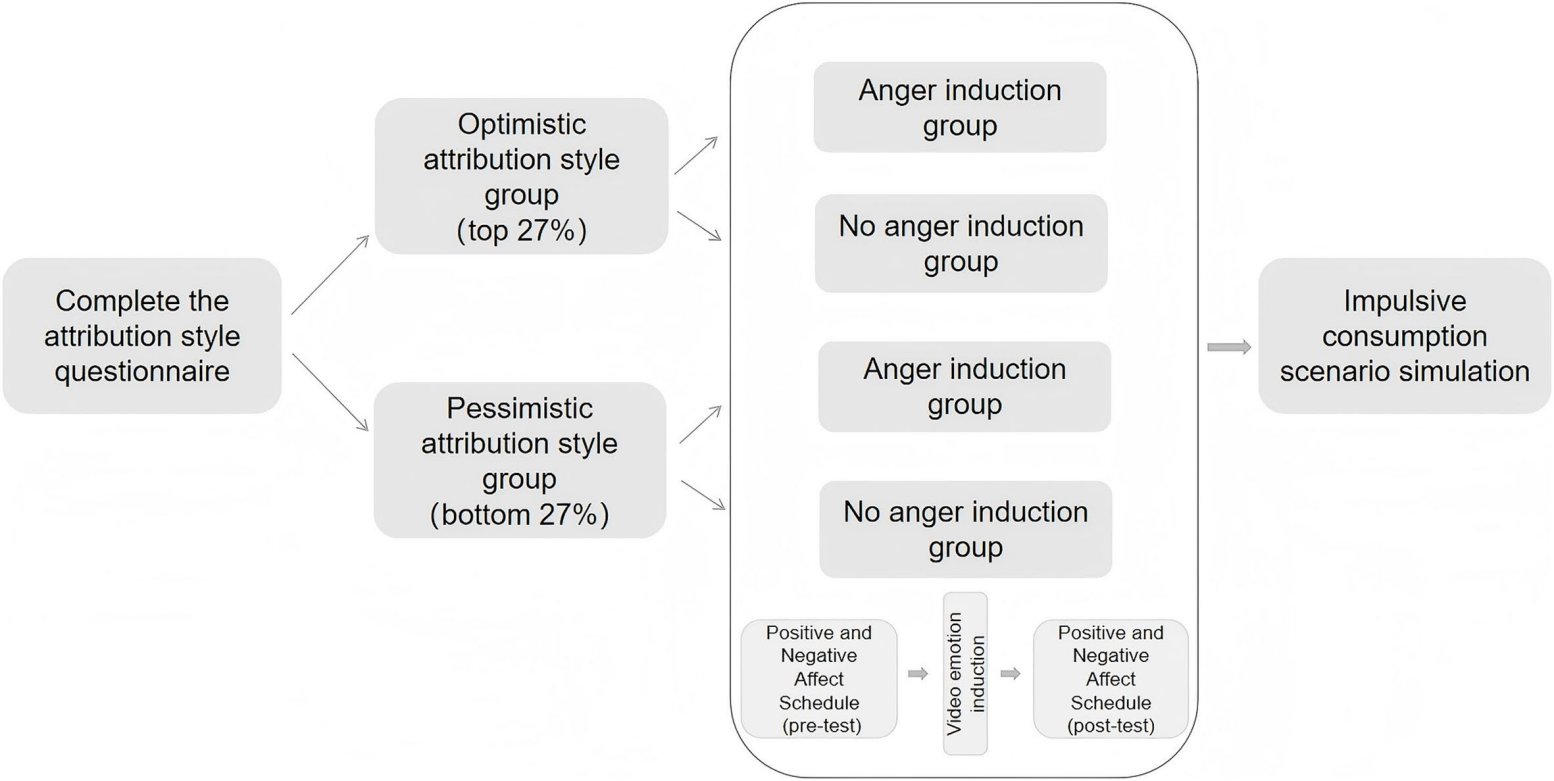
Experimental procedure of Experiment 2.

Immediately after viewing, participants completed the Emotional Self-Assessment Scale again as a post-test assessment. Subsequently, all participants engaged in three standardized impulse consumption scenarios, with their impulsive consumption levels quantified using validated scoring metrics (Figure 3).

After completing the experiment, participants underwent a structured emotional recovery protocol, including a standardized debriefing session that explained the purpose of the emotion induction and its temporary nature. Participants were then exposed to several minutes of upbeat, relaxing music. Thereafter, they were monitored for signs of residual distress and received a small gift as a token of appreciation. The participants were explicitly informed that they would be provided immediate, on-site psychological support services available at no cost, should they experience residual discomfort. The experimental procedure, including the specific use of the anger-inducing stimulus, was reviewed and approved by the ethics committee of Hunan Normal University, which confirmed that all measures were in strict compliance with ethical guidelines for the protection of human participants.

#### 3.2.5 Data analysis

Two-way ANOVA was used to analyze the effects of attributional style and anger on impulsive consumption. Simple effects tests were used to examine interaction patterns.

### 3.3 Results

Statistical analyses of the participants’ emotional valence scores before and after video exposure were conducted to validate the efficacy of emotion induction (Tables 1, 2).

**Table 1:** Participants’ emotional changes in the no anger induced group before and after watching the video.

Emotions	Pre-test and post-test (*Mean ± Standard deviation*)		*t*	*p*
	Before watching (*n* = 60)	After watching (*n* = 60)		
Happiness	2.88 ± 1.06	2.63 ± 1.38	1.870	0.066
Surprise	2.35 ± 1.30	2.07 ± 1.29	1.775	0.081
Fear	1.87 ± 1.21	1.72 ± 1.17	1.321	0.192
Disgust	1.57 ± 1.03	1.77 ± 1.16	−1.387	0.171
Sadness	1.95 ± 1.35	1.90 ± 1.34	0.375	0.709
Anger	1.63 ± 1.04	1.65 ± 1.09	−0.136	0.892
Calmness	3.48 ± 1.16	3.62 ± 1.22	−0.861	0.393

**Table 2:** Participants’ emotional changes in the anger-induced group before and after watching the video.

Emotion	Pre-test and post-test (*Mean ± Standard deviation)*		*t*	*p*
	Before watching (*n* = 60)	After watching (*n* = 60)		
Happiness	2.70 ± 1.42	1.42 ± 0.96	8.131	<0.001
Surprise	2.15 ± 1.10	2.12 ± 1.33	0.214	0.831
Fear	1.75 ± 1.00	2.23 ± 1.23	−3.236	0.002
Disgust	2.00 ± 1.35	3.60 ± 1.53	−7.010	<0.001
Sadness	2.17 ± 1.39	3.85 ± 1.29	−7.659	<0.001
Anger	2.03 ± 1.38	3.98 ± 1.35	−8.774	<0.001
Calmness	3.13 ± 1.32	1.98 ± 1.10	5.415	<0.001

Table 1 demonstrates that the participants in the neutral (no anger induction) condition exhibited no significant changes in emotional scores across all dimensions after viewing the video (*p* > 0.05).

Table 2 reveals significant post-induction changes in emotional scores for the anger group (*p* < 0.01) across all dimensions except surprise (*p* = 0.831). Specifically, participants in the anger-induction condition showed marked reductions in joy and calmness and significant increases in anger, disgust, sadness, and fear.

**Figure 4:**
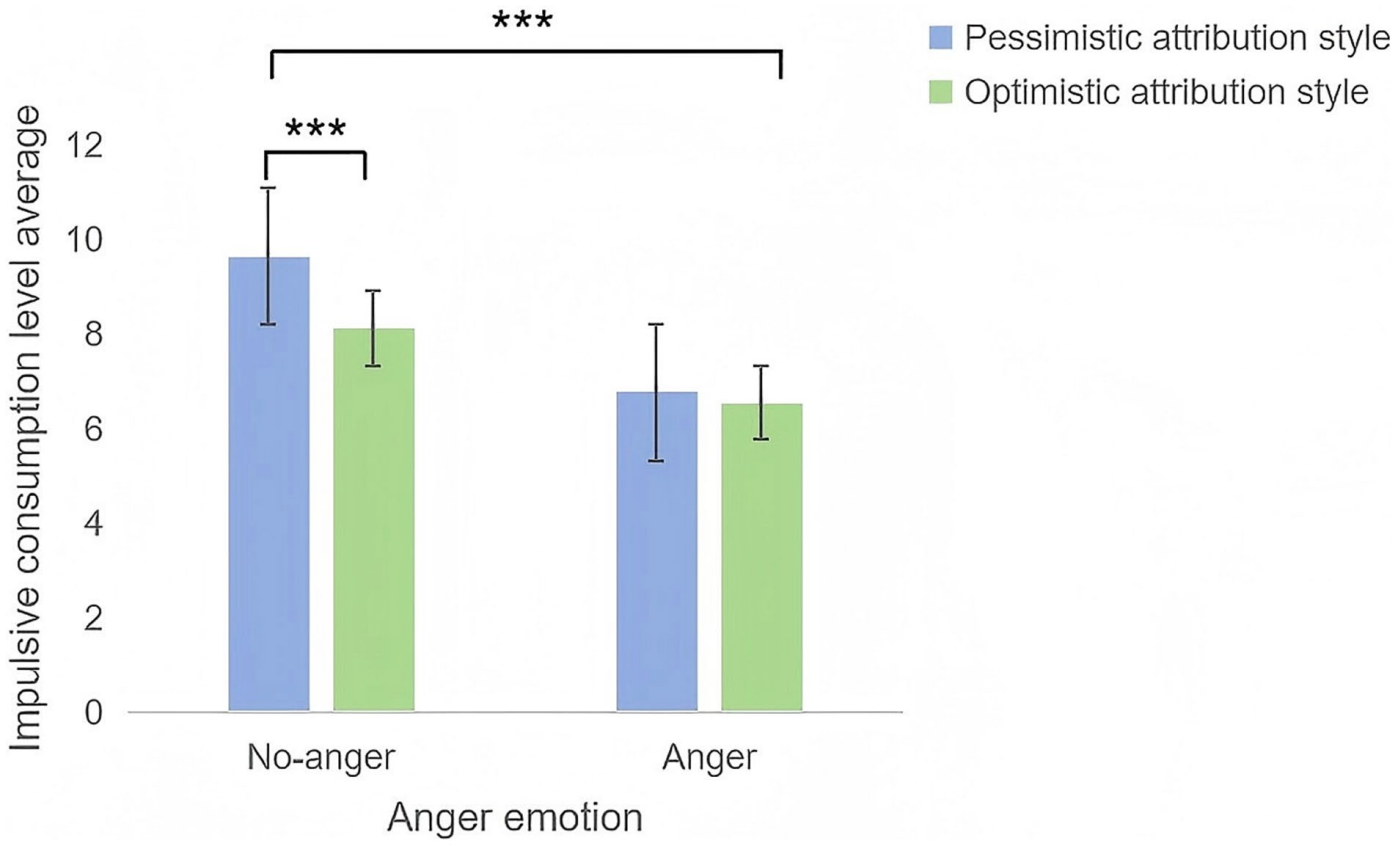
Mean impulsive consumption scores across attributional styles and anger conditions.

A two-way ANOVA with impulsive consumption as the dependent variable (2 attributional styles × 2 anger conditions) yielded the following results (Figure 4).

Main effect of attributional style: *F*(1, 116) = 11.977, *p* = 0.001, η^2^ = 0.094, indicating significant differences in impulsive consumption between optimistic and pessimistic groups;

Main effect of anger: *F*(1, 116) = 59.456, *p* < 0.001, η^2^ = 0.339, reflecting significantly higher impulsive consumption in the neutral condition than under anger induction;

Interaction effect: *F*(1, 116) = 4.516, *p* = 0.036, η^2^ = 0.037. This suggests that while anger induction moderated the relationship between attributional style and impulsive consumption, the effect was modest in magnitude.

Simple effects analysis (Figure 4) was used to further clarify these patterns. Pessimistic group: participants in the neutral condition scored significantly higher on impulsive consumption than those under anger induction (p < 0.001). Optimistic group: Similarly, the neutral condition participants exhibited higher impulsivity than the anger induction participants (p < 0.001). Neutral condition: Pessimistic individuals demonstrated significantly higher impulsive consumption than optimistic individuals (p < 0.001);

Anger induction: No significant differences emerged between the attributional styles (*p* = 0.347).

### 3.4 Discussion

These findings confirm the effectiveness of the emotional manipulation, with participants in the anger-induction group displaying significantly heightened anger levels following stimulus exposure. Notably, the induction also led to increases in other negative emotions such as fear, disgust, and sadness—consistent with the complex and multifaceted nature of responses to traumatic historical footage. Therefore, the observed inhibitory effects on impulsive consumption might be attributed to the general activation of high-arousal negative affect, rather than to the unique effect of anger, which is a limitation of the current study. This also warrants that future investigations use more specific induction paradigms (e.g., recalling personal angering events) or physiological measures to better understand the unique influences of discrete negative emotions on consumer decision-making.

These results support Hypothesis 2, indicating that anger induction exerts inhibitory effects on impulsive consumption—which contradicts some prior work linking anger to increased impulsivity (e.g., Rook, 1987; Redine et al., 2023). However, this discrepancy may be reconciled by considering the nature of the anger induction and its consequent cognitive appraisals. Our paradigm utilized a film clip depicting historical injustices, which may have induced a more complex, morally laden form of anger. According to cognitive appraisal theories (Lerner and Keltner, 2000), such anger can be characterized by a high sense of certainty and individual control, potentially leading to more deliberative (rather than impulsive) decision-making as a form of regained autonomy. This enhanced deliberative processing likely involves the recruitment of neural circuits associated with cognitive control, such as the dorsolateral prefrontal cortex (dlPFC), which is critical for suppressing dominant impulsive responses (Hare et al., 2009; Disner et al., 2011). The appraisals of certainty and control may thus facilitate top-down regulation from these prefrontal regions, thereby curbing purchase impulses. Therefore, the anger induced in our study may have amplified the participants’ cognitive restraint capacity, thereby curbing purchase impulses. This pattern aligns with emerging theoretical propositions regarding anger’s nuanced regulatory role in self-control processes (Koley and Warren, 2025).

However, it is important to note that the interaction effect between attributional style and anger, while statistically significant, was small in magnitude (partial η^2^ = 0.037). This indicates that while the pattern of our results is reliable, its practical significance and influence in real-world settings may be limited. The boundaries of this effect—such as under what conditions it is most pronounced—require further clarification. As such, future research with larger samples should investigate potential moderating variables (e.g., gender, socioeconomic status, or monthly consumption level) that might strengthen or weaken this interaction, thereby better defining its applicable scope.

Nevertheless, the current findings should be interpreted within the broader context of metacognitive influences on impulsive consumption, as emphasized by Verhagen and van Dolen (2011). Critical questions remain regarding how self-regulatory mechanisms interact with attributional biases to modulate emotional impacts on purchasing behaviors. Subsequent analyses employing impulse consumption simulations should systematically investigate the tripartite relationships among attributional tendencies, self-control thresholds, and affective states. This approach disentangles the relative contributions of emotional and cognitive components in shaping university students’ consumption patterns.

## 4 Experiment 3: Interactive effects of attributional style and self-control on college students’ impulsive consumption

### 4.1 Research purpose and hypotheses

This experiment examined the combined effects of attributional style and self-control on impulsive consumption using simulated purchasing scenarios. The hypotheses were as follows:

*Hypothesis 4:* Self-control significantly influences impulsive consumption; thus students with low self-control exhibit higher impulsive consumption than those with high self-control.

*Hypothesis 5:* Attributional style and self-control interact to influence impulsive consumption; thus, pessimistic individuals with low self-control exhibit the highest impulsive consumption, whereas optimistic individuals with high self-control exhibit the lowest impulsive consumption.

### 4.2 Methodology

#### 4.2.1 Participants

A power analysis (G*Power 3.1; Faul et al., 2007) determined a required sample size of 201 participants for 80% power (1–β) with α = 0.05 and a medium effect size (*f* = 0.25). Using convenience sampling, 248 undergraduate students were recruited from a university in Changsha, China. After the exclusion of 26 invalid responses (incomplete or inconsistent answers), 222 valid datasets were retained. Participants who scored in the top and bottom 27% of the ASQ were classified as optimistic (n = 60) or pessimistic (n = 60), respectively. Each group was further divided into high (n = 30) and low (n = 30) self-control subgroups based on Self-Control Scale scores, yielding 120 participants (53 female; *M_age_* = 18.4 years, *SD* = 0.76). All participants met the same health, linguistic, and procedural criteria as in the previous experiments. All signed informed consent forms.

#### 4.2.2 Experimental design

A 2 (attributional style: optimistic versus pessimistic) × 2 (self-control: high versus low) between-subjects design was employed. The independent variables included attributional style (ASQ scores) and self-control (Self-Control Scale scores), with impulsive consumption level as the dependent variable.

#### 4.2.3 Experimental materials

##### 4.2.3.1 ASQ

These were the same as those employed in Experiment 1.

##### 4.2.3.2 Impulsive consumption scenario simulation

These were the same as those employed in Experiment 1.

##### 4.2.3.3 Self-control scale

Adapted from Mamayek et al. (2017) and revised by Tan and Guo (2008), this 19-item scale assesses 5 dimensions of the self-control trait on a 5-point Likert scale. Lower total scores indicate stronger self-control. The Chinese adaptation demonstrated strong reliability (Cronbach’s α = 0.86) and cultural validity (Bian et al., 2025). For the purpose of data analysis and to facilitate intuitive interpretation, the raw scores were reverse-scored prior to grouping. Consequently, higher scores on the transformed scale correspond to higher levels of self-control, and the groups were labeled as “high self-control” and “low self-control” based on these reversed scores.

#### 4.2.4 Procedure

**Figure 5:**
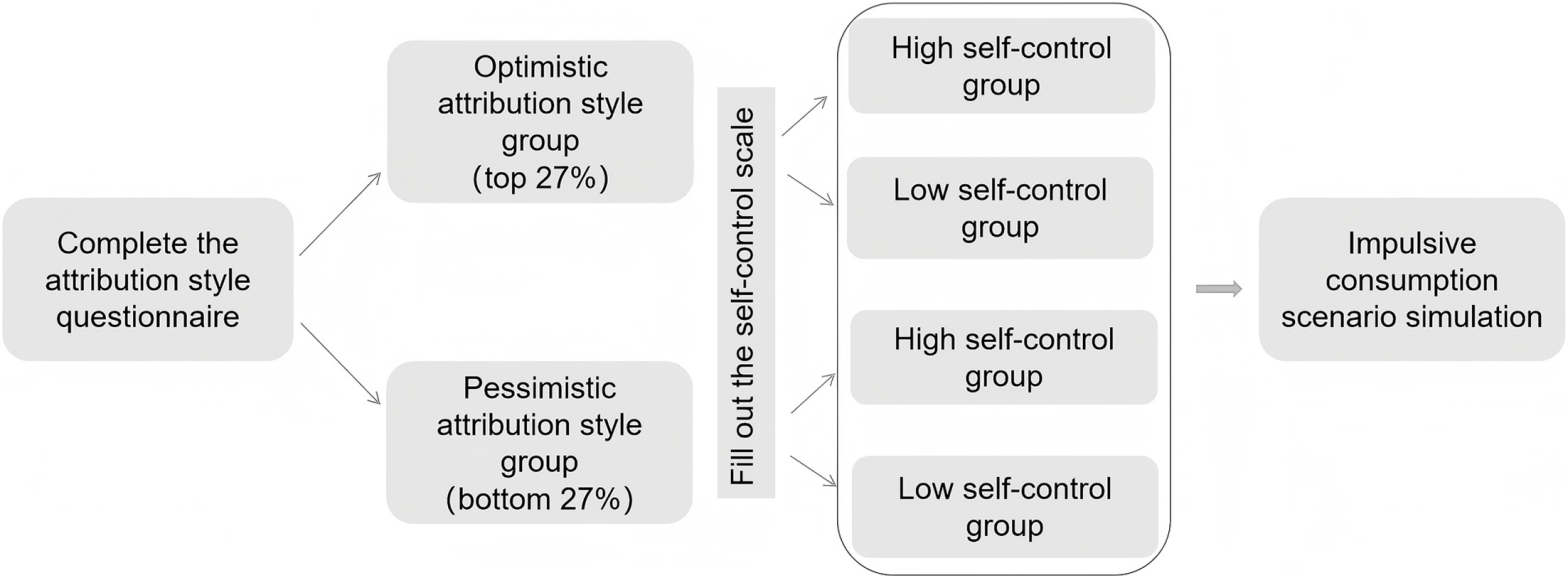
Experimental procedure of Experiment 3.

Participants first completed the ASQ and were then classified into optimistic (n = 60) or pessimistic (n = 60) groups. Subsequently, based on the Self-Control Scale score, each group was divided into high (n = 30) and low (n = 30) self-control subgroups. Participants completed three impulsive consumption scenarios with scores indexing impulsivity (Figure 5). This was approved by the ethics committee.

#### 4.2.5 Data analysis

Two-way ANOVA was used to analyze the effects of attributional style and self-control on impulsive consumption. Simple effects tests were used to examine interaction patterns.

### 4.3 Results

**Figure 6:**
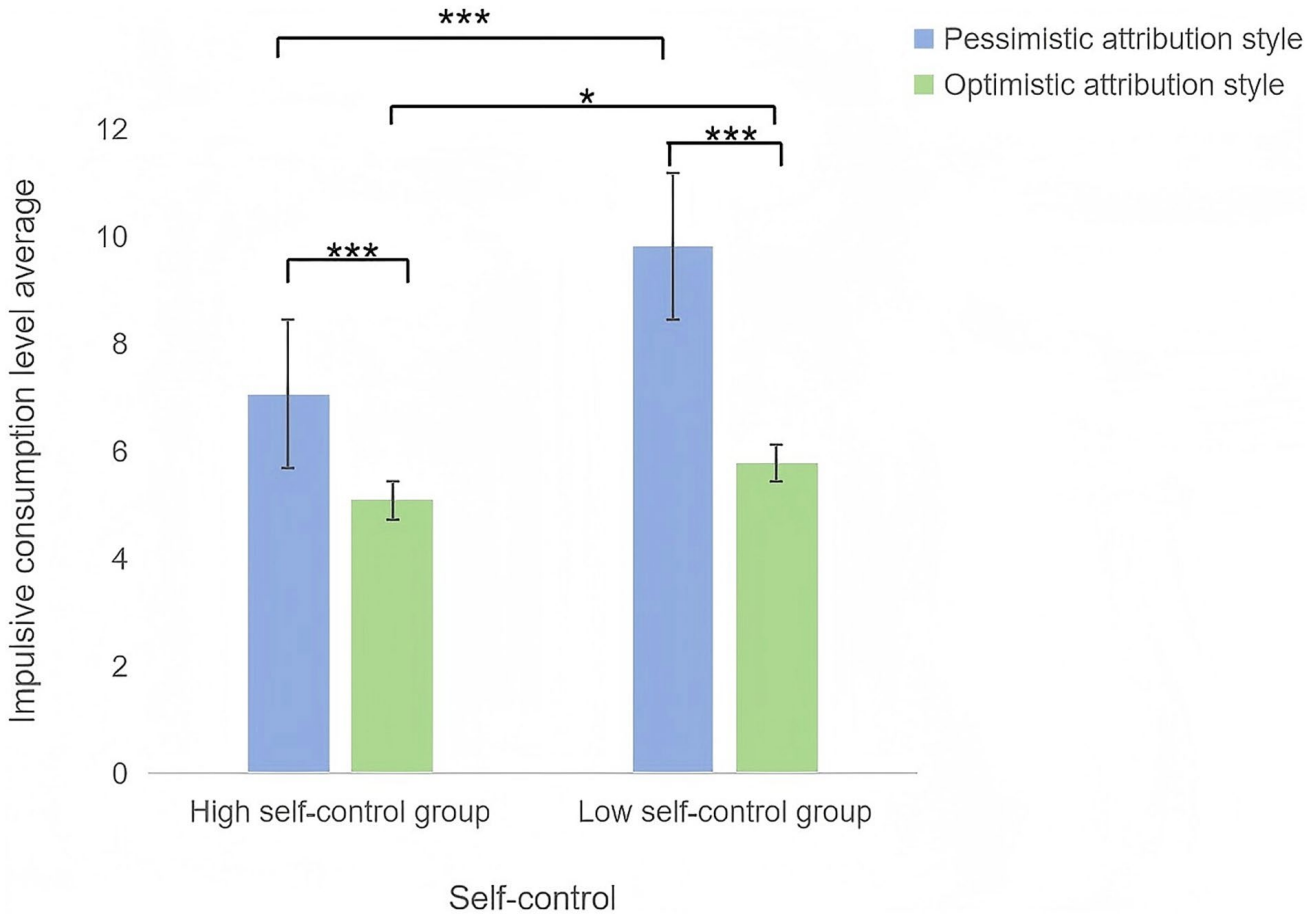
Mean impulsive consumption scores across attributional styles and self-control levels.

A two-way ANOVA (two attributional styles × two self-control levels), with impulsive consumption as the dependent variable, yielded the following results: (Figure 6).

Main effect of attributional style: *F*(1, 116) = 169.561, *p* < 0.001, η^2^ = 0.594, indicating significant differences in impulsive consumption between optimistic and pessimistic attributional styles.

Main effect of self-control: *F*(1, 116) = 55.429, *p* < 0.001, η^2^ = 0.323, reflecting significantly higher impulsive consumption in participants with low self-control compared to those with high self-control.

Interaction effect: *F*(1, 116) = 19.747, *p* < 0.001, η^2^ = 0.145, demonstrating that the influence of attributional style on impulsive consumption varied depending on self-control levels.

Simple effects analysis was used to further clarify these patterns. Pessimistic group: participants with low self-control exhibited significantly higher impulsive consumption than those with high self-control (*p* < 0.001). Optimistic group: similarly, low self-control participants scored higher in impulsivity than high self-control participants (*p* < 0.05). Low self-control: pessimistic individuals demonstrated significantly higher impulsive consumption than optimistic individuals (*p* < 0.001). High self-control: despite higher self-control, pessimistic participants still showed greater impulsivity than optimistic participants (*p* < 0.001).

### 4.4 Discussion

Experiment 3 confirmed that both attributional style and self-control significantly influenced impulsive consumption. Optimistic individuals exhibited lower impulsivity than pessimistic individuals, consistent with Experiment 1. Low self-control predicted higher impulsive consumption, supporting Hypothesis 4 and echoing Vohs and Faber’s (2007) findings on self-control resource depletion. The interaction effect revealed that pessimistic individuals with low self-control displayed the highest impulsivity, whereas optimistic individuals with high self-control showed the lowest, validating Hypothesis 5. These results underscore the synergistic roles of cognitive framing (attributional style) and regulatory capacity (self-control) in impulsive decision making.

## 5 General discussion

### 5.1 Optimistic attributional style significantly inhibits college students’ impulsive consumption

This study confirms the significant influence of attributional style on impulsive consumption, with pessimistic individuals exhibiting higher impulsivity and optimists demonstrating more rational decision making. These findings align with the attribution theory (Weiner, 1985; Sanders, 2024), which posits that explanatory styles shape subsequent behaviors, and with self-regulation theory (Carver and Scheier, 2001), which suggests that optimistic attributional styles enhance self-control to curb impulsive consumption (Baumeister et al., 2007). Furthermore, the results extend the cognitive-affective personality system theory (Mischel and Shoda, 1995) by revealing how attributional style, as a stable cognitive predisposition, modulates emotional regulation and decision evaluation to influence consumption behaviors.

This study challenges the traditional overemphasis on situational factors in consumer behavior research (Rook, 1987; Redine et al., 2023), highlighting instead the critical role of individual cognitive differences. Pessimistic individuals, who tend to attribute negative events to stable, global causes (Abramson et al., 1978), may adopt a “what-the-hell” mentality (Puri, 1996) when confronted with promotions or temptations, exacerbating impulsive spending. Conversely, optimists employ problem-focused strategies (Folkman and Lazarus, 1988), weighing long-term consequences to reduce irrational expenditures. These insights enrich our understanding of the cognitive mechanisms underlying impulsive consumption and provide a novel theoretical framework for exploring the relationships between attributional style and other consumption biases (e.g., compulsive buying and conspicuous consumption). Practically, the findings on the role of attributional style may inform the development of targeted interventions. Evidence-based approaches such as attributional retraining, which has proven effective in improving academic outcomes and mental health by modifying maladaptive explanatory styles (Kabat-Zinn, 2003; Seligman et al., 1999), could be explored as a potential strategy to foster adaptive cognitive patterns that may reduce impulsive consumption tendencies. Similarly, integrating assessments of attributional style into financial literacy programs (Bajtelsmit, 2024) could allow for more personalized guidance. However, the efficacy of these specific applications for reducing impulsive spending requires direct empirical testing in future intervention studies. A specific ART module might involve: (1) psychoeducation: helping students identify their own pessimistic attributional patterns (e.g., “I always fail to control my spending”) through guided self-assessment; (2) cognitive restructuring: teaching students to challenge these patterns and adopt a more flexible, optimistic style (e.g., “that advertisement was designed to be tempting, but I can make a conscious choice”); and (3) behavioral experimentation: using role-playing or simulated shopping scenarios to practice new attributional styles and strengthen adaptive cognitive habits. This structured approach moves beyond general advice to provide actionable skills for improving self-regulation in consumer contexts.

### 5.2 Anger induction suppresses college students’ impulsive consumption

This study elucidates the complex interplay between attributional style and anger in shaping impulsive consumption. Under neutral conditions, pessimistic individuals exhibited the highest impulsivity, while optimists showed greater restraint, a pattern consistent with attribution theory (Weiner, 1985; Sanders, 2024) and the role of optimism in promoting self-efficacy and future-oriented thinking (Peterson and Seligman, 1984). However, anger induction eliminated these differences, suggesting that anger temporarily overrides stable attributional effects via emotion-cognition integration mechanisms (Lerner and Keltner, 2000). These results challenge conventional emotion decision theories by demonstrating the unexpected inhibitory effects of anger. While anger typically activates behavioral approach systems (Harmon-Jones et al., 2013), it paradoxically enhanced cognitive control in this context, possibly by increasing perceived agency (Lerner and Tiedens, 2006) or narrowing attentional focus (Gable and Harmon-Jones, 2010). This underscores the need to differentiate motivational dimensions of anger (e.g., directed vs. diffuse; Carver and Harmon-Jones, 2009) in future research. Intervention strategies should integrate cognitive restructuring (Schaeuffele et al., 2024) and emotion regulation training (Gross, 2015) for pessimistic individuals while fostering emotion-cognition awareness (Salovey et al., 2009) across student populations. Scenario-based simulations (Loewenstein, 2005) could further help students practice rational decision making under emotional arousal. It is important to note that although using anger induction was effective, it co-activated other negative emotions such as fear and disgust. Thus, the suppression of impulsive consumption may reflect a broader response to high-arousal negative states besides general anger. Future research should aim to isolate the unique cognitive and motivational aspects of anger (e.g., sense of certainty, approach motivation) using more targeted manipulations to clarify its distinct role in consumption behavior.

### 5.3 High self-control effectively mitigates college students’ impulsive consumption

This study revealed the synergistic mechanisms between attributional style and self-control in impulsive consumption. Optimistic attributional styles buffered impulsivity significantly, with protective effects varying across self-control levels. These findings refine the social cognitive theory (Bandura, 2023) by demonstrating how attributional styles modulate self-control resource allocation. Optimists maintained rational decision-making even under self-control depletion, challenging the universality of ego depletion theory (Baumeister et al., 2007) and supporting control-process theory (Fujita et al., 2024) on goal clarity. The results advocate for a tripartite intervention framework: (1) Tiered interventions: combining cognitive restructuring and executive function training for high-risk students (low self-control + pessimistic attribution; Duckworth et al., 2016); (2) Attributional training: integrating growth mindset cultivation (Dweck, 2006) and metacognitive skill development into curricula; and (3) Environment redesign: applying behavioral economics principles (Leonard, 2008) to implement decision prompts and delay mechanisms. Universities should prioritize three core competencies: rational attribution, impulse control, and emotion regulation, adopting a “pilot-refine-scale” strategy for program implementation.

### 5.4 Limitations

The study sample was confined to students aged 17–22 years from a single university in Changsha, which may limit the generalizability of our findings. Regional and cultural specificities could affect the applicability of the results to broader student populations. Future research should expand the sample to include students from diverse geographical regions and higher education institutions to enhance the external validity of the findings. Furthermore, the reliance on self-reported responses to hypothetical third-person scenarios, while methodologically convenient, may limit the generalizability of findings to actual purchasing behavior. Future studies could employ more ecologically valid measures, such as behavioral economic tasks involving real monetary incentives or first-person virtual shopping simulations, to corroborate our findings. This study employed two separate 2 × 2 between-subject designs to examine variable relationships. Future studies could adopt a 2 × 2 × 2 mixed-design to simultaneously investigate the interaction effects of three variables, and utilize longitudinal tracking studies to elucidate the dynamic processes underlying causal relationships between variables. Furthermore, the gender distribution across our experiments was uneven. This imbalance may limit the generalizability of our findings and potentially confound the observed effects, as prior empirical theory suggests the prevalence of significant gender differences in impulsive consumption behavior. Future studies should strive for a more balanced gender representation to explicitly examine and control for the influence of this variable. Additionally, employing physiological measures (e.g., skin conductance response, heart rate variability) could provide objective, convergent evidence to robustly support the efficacy of the emotional induction procedures used in such research, and move beyond self-report measures.

## 6 Conclusion

College students’ impulsive consumption exhibits significant attributional style differences: optimists suppress impulsivity, while pessimists are more prone to impulsive behaviors; anger induction amplifies these attributional differences, highlighting the emotion’s critical moderating role in consumption decisions; and high self-control effectively buffers the negative impact of pessimistic attributional styles on impulsive consumption.

## Data Availability

The raw data supporting the conclusions of this article will be made available by the authors, without undue reservation.
